# The Impact of Genetic Polymorphisms on the Clinical Efficacy of Azole Antifungals

**DOI:** 10.3390/genes16091058

**Published:** 2025-09-09

**Authors:** Hareesh Singam, Sherif Mossad

**Affiliations:** Department of Infectious Diseases, Section of Transplant Infectious Diseases, Cleveland Clinic Foundation, Cleveland, OH 44195, USA; mossads@ccf.org

**Keywords:** CYP2C19, therapeutic drug monitoring, voriconazole, posaconazole, itraconazole, cytochrome P450

## Abstract

Azoles are the primary agents for antifungal activity in clinical medicine due to their broad-spectrum efficacy and favorable safety profiles compared to older agents. Triazoles, including fluconazole, itraconazole, voriconazole, posaconazole, and isavuconazole, have varied pharmacokinetic and pharmacodynamic properties. This is due to various polymorphisms in hepatic enzymes, necessitating genotype-guided dosing and therapeutic drug monitoring (TDM) to optimize treatment outcomes. This review highlights the clinical relevance of pharmacogenomics in azole therapy, particularly the role of cytochrome P450 (CYP450) enzyme polymorphisms in influencing drug levels, efficacy, and toxicity. Understanding these genetic and metabolic factors is essential for personalized antifungal treatment strategies, improving patient safety and therapeutic outcomes.

## 1. Introduction

Azoles are the most commonly and widely used antifungals in clinical medicine due to their efficacy regarding clinically relevant fungi and their favorable safety profiles compared to the polyene antifungal amphotericin B. Azoles are composed of two classes of drugs, imidazoles and triazoles, based on the number of nitrogen atoms in their azole rings. Imidazoles have two nitrogen azole rings and are used topically. Examples of these include ketoconazole, miconazole, and clotrimazole [1]. It is important to note that oral ketoconazole can be used for systemic therapy but has a Food and Drug Administration (FDA) black-box warning for fatal hepatotoxicity, QT prolongation, adrenal insufficiency, and drug–drug interactions. Therefore, it is used if no alternative agent is available [2]. Triazoles have three nitrogens in their azole ring and are used systemically. Triazoles include fluconazole, itraconazole, voriconazole, and posaconazole [3]. Fluconazole was initially developed but had a narrow spectrum of activity against molds—specifically against *Aspergillus*. Itraconazole has FDA approval for several anti-mold infections, but, to expand the therapeutic options, voriconazole, posaconazole, and isavuconazole were also developed.

There has been a steady increase in the use of triazoles in clinical practice over the last two decades. This has been due to several factors, including more complex patients needing intensive care, an increase in oncological treatments, hematopoietic and organ transplants becoming more common, and increasing rates of invasive fungal infections in certain endemic areas. All of these factors have contributed to the increased use of antifungal drugs [4]. The first systemic antifungal developed, amphoteracin B, has broad-spectrum antifungal activity but significant side effects, particularly nephrotoxicity, which also contributed to the development of the azole class of antifungals.

There are a variety of factors that impact the absorption, distribution, and metabolism of azoles. These include the gastric pH, which varies widely within populations, impacting the absorption of posaconazole (where an increased gastric pH and less acidic environment reduce absorption) and itraconazole (decreased gastric pH and more acidic environment increase absorption) [5]. In addition, the method of drug delivery also contributes to absorption. The solution form of itraconazole is better absorbed than the oral tablet, but its use is limited due to its poor taste and smell. Serum binding is another factor, since itraconazole and posaconazole are more protein-bound than fluconazole and voriconazole. Therefore, variability in the levels of serum albumin can affect their bioavailability and thus their efficacy [6].

Most drugs, including azoles, are taken into cells by passive diffusion and by active transport. Specific transporters have significant genetic variants, which often vary based on ethnicity. Azoles act as substrates or inhibitors of these transporters [5]. In addition, orally administered drugs undergo first-pass metabolism, often by the cytochrome P450 enzyme system.

This is a phase I oxidative metabolic process, which most drugs, including azoles, undergo in the liver and the gastrointestinal tract [5]. Azoles bind to the heme centers of these enzymes, inhibiting their function [7]. As such, this inhibition leads to higher levels of drugs that are co-administered. In addition, the activity of CYP enzymes and their isozymes varies with age, gender, and ethnicity. These variations can lead to variability in the response to and/or toxicity of these drugs [8].

Cytochrome P450 enzymes are classified based on their similar gene sequences (e.g., *CYP1*, *CYP2*) and a subfamily letter (e.g., *CYP1A*, *CYP2D*) [9]. Drugs with a common pathway lead to drug–drug interactions. In the human liver, the most common pathways include CYP3A4/5, CYP2C9, CYP2D6, and CYP2C19. The structural differences between these enzymes confer selectivity for substrates and sites of metabolism for shared substrates. For example, the crystal structures of CYP2C8 and CYP2C9 have extensive amino acid differences for side chains. This shape of the substrate-binding cavities leads to differences in their substrate and inhibitor profiles [10].

The main objective of this review is to examine the impact of genetic polymorphisms, particularly CYP450 enzymes and their isozymes, on azoles’ antifungal efficacy and clinical outcomes.

## 2. Mechanisms of Action and Pharmacokinetics of Azole Antifungals

Both classes of azoles, imidazoles and triazoles, work by competitively inhibiting the fungal CYP51-class cytochrome P450 superfamily enzyme 14α-sterol demethylase. The CYP51 enzymes are vital for the synthesis of ergosterol, a component of the plasma membrane in most fungi [11]. Inhibition of this enzyme leads to the accumulation of 14-α-methylsterols on the fungal surface, which results in the arrest of fungal growth, and they are thus generally considered fungistatic rather than fungicidal [3].

Azoles require hepatic metabolism by CYP enzymes to be eliminated from the human body. The biliary route is the major excretory pathway (>80%), and 20% of the metabolites are eliminated in the urine [6]. The rate of elimination is dose-dependent and biphasic. Thus, the greater the dose, the longer the half-life for drug elimination (average of around 30 h) [12]. Rapid elimination occurs in the first two hours, followed by a slower decline over the next 6–9 h. This long half-life and fungistatic mechanism of action result in a longer time period required for efficacy [6].

Recently, super-bioavailable (SUBA) technology has been utilized to enhance absorption by using a pioneering spray-drying technique to create an amorphous solid dispersion. This is achieved by combining the drug with a pH-dependent drug polymer matrix, which reduces its solubility in the acidic environment of the stomach. For example, a newer formulation of itraconazole using SUBA (brand name: Tolsura) is absorbed in the duodenum rather than the stomach [13].

## 3. CYP2C19 Polymorphisms and Voriconazole

Voriconazole has a broad spectrum of activity against several clinically significant fungi, such as fluconazole-resistant *Candida* species, *Aspergillus*, *Fusarium*, and *Scedosporium*. Voriconazole is mainly metabolized by CYP2C19 and CYP3A4 [14]. Several other drugs are also metabolized by the same metabolic pathways, leading to bidirectional drug interactions, resulting in the inhibition or induction of the metabolism of voriconazole, as well as the metabolism of these drugs. These pharmacokinetics are influenced by the underlying *CYP2C19* genotype. It is important to note that the affinity of voriconazole to CYP3A4 is about 50-fold lower than to CYP2C19. Therefore, assessing *CYP2C19* polymorphisms is very important [14].

Variations in *CYP2C19* polymorphisms are based on ethnicity. Approximately 15 to 20% of Asian populations are expected to be poor metabolizers, while only 3 to 5% of Caucasian and Black individuals are poor metabolizers [15]. Voriconazole polymorphism commercial assays are only available at a few tertiary care centers and reference laboratories.

Several studies indicate that the enteral administration of voriconazole leads to rapid absorption, with almost 100% bioavailability, but this does not account for *CYP2C19* genetic polymorphisms [16]. Several variants of *CYP2C19*, such as CYP2C19*1/*1, CYP2C19*1/*2, and *1/*3, have been associated with the rapid metabolism of voriconazole, while others, such as CYP2C19*2/*2, *2/*3, and *3/*3, have been associated with the poor metabolism of voriconazole [14]. In a study by Scholz et al., the oral bioavailability of voriconazole was 94.4% in CYP2C19 poor metabolizers and 75.2% in rapid metabolizers.

Therefore, the TDM of voriconazole has become the standard of care to ensure efficacy and avoid adverse effects. Low serum voriconazole levels are associated with treatment failure, leading to uncontrolled and potentially fatal invasive mold infections, while elevated serum voriconazole levels are often but not always associated with adverse effects. These include short-term side effects such as hepatotoxicity, visual hallucinations, and prolongation of the QTc interval. Long-term effects of voriconazole include photosensitivity (which is a risk factor for cutaneous squamous cell carcinomas), fluoride accumulation in the bone matrix (resulting in periostitis), peripheral neuropathy, alopecia, nail changes, and others [17]. It is important to note that not all side effects, such as visual hallucinations, need discontinuation or dose modifications, and they often self-resolve with continued therapy.

The Clinical Pharmacogenetics Implementation Consortium (CPIC^®^) Guideline for *CYP2C19* and Voriconazole Therapy, published in 2016, can be used for guidance in voriconazole dosing [18]. This further expands on the CYP2C19 phenotypes to include the CYP2C19 ultrarapid metabolizer (*17/*17), CYP2C19 rapid metabolizer (*1/*17), CYP2C19 normal metabolizer (*1/*1), CYP2C19 intermediate metabolizer (*1/*2, *1/*3, *2/*17), and CYP2C19 poor metabolizer (*2/*2, *2/*3, *3/*3). Table 1 summarizes the recommended dosing of voriconazole based on this guideline.

Studies have shown that voriconazole trough concentrations ≥ 1.0 mg/L are associated with treatment success. Supratherapeutic concentrations were associated with neurotoxicity and hepatotoxicity. These associations were more prevalent in Asian populations. A meta-analysis of 20 studies showed that poor metabolizers taking voriconazole were at an increased risk of overall adverse events compared to normal or intermediate metabolizers [19]. Other studies failed to show such associations.

*CYP2C19* polymorphisms significantly affect voriconazole metabolism. Genotype-guided dosing with TDM was reported to increase the likelihood of achieving a therapeutic range [20]. Besides *CYP2C19*, other genetic variants, e.g., *CYP3A4*, *CYP2C9*, *ABCB1*, *ABCG2*, *ABCC2*, *SLCO1B3*, *SLCO2B1*, *FMO3*, *NR1I2*, and *POR*, can impact voriconazole levels as well [19].

## 4. Genetic Variability of Other Azole Antifungals

Fluconazole

Fluconazole is considered the first-line therapy against many Candida species, including *Candida albicans*, *Candida parapsilosis*, *Candida tropicalis*, *Candida lusitaniae*, and *Candida dubliniensis*, but has variable activity against *C. glabrata* and is often not active against *Candida krusei*. Fluconazole displays excellent activity against *Cryptococcus neoformans* and several dimorphic pathogens, including *Blastomycosis dermatitidis*, *Coccidioides immitis*, and *Histoplasma capsulatum* [21].

Fluconazole inhibits several CYP enzymes, including 2C9, 2C19, and, to a lesser extent, 3A4, but not CYP1A2, 2A6, or 2E1 [5,22]. These enzymes are the primary mediators of fluconazole drug interactions. In addition to being metabolized by CYP enzymes, fluconazole is also metabolized by uridine diphosphate (UDP) glucuronosyltransferases (UGT)—more specifically, the UGT2B7 isoform. This is a family of enzymes that detoxify compounds by generating products that are more polar and, thus, more readily excreted in the bile or urine [23].

Fluconazole is both a substrate and an inhibitor of UGT2B7, which converts fluconazole to the urinary metabolite fluconazole glucuronide [5,23,24]. However, the effects of CYP and drug transporters on fluconazole metabolism are limited (Table 2). Therefore, their genetic variation has relatively little impact on fluconazole pharmacokinetics, unlike for voriconazole [5]. Studies have shown that fluconazole levels in critically ill patients are primarily impacted by the size of the administered dose, augmented renal clearance, and renal replacement therapy [25].

Itraconazole

Itraconazole is FDA-approved for blastomycosis, histoplasmosis, and aspergillosis. It has also shown efficacy in treating paracoccidioidomycosis, coccidioidomycosis, talaromycosis, and candidiasis [30]. Itraconazole is a highly lipophilic, slightly acidic salt molecule. Therefore, it is ionizable only at very low pH values. It is also very poorly soluble in water [29,31]. Hence, the oral absorption of itraconazole is limited when it is administered as capsules, with reduced bioavailability. It is recommended to give capsules together with food and with acidic fluids such as cola drinks to improve the oral bioavailability, as stated earlier.

In the body, itraconazole is metabolized into more than 30 metabolites, the most important of which is hydroxyitraconazole. It is primarily metabolized by the CYP450 system; the main isoenzymes involved are CYP3A4, CYP2C9, or CYP2C19 [29]. In addition, a half-life of 20 h has been reported for itraconazole after the oral administration of a single 200 mg dose. As with other azoles, itraconazole is a potent enzymic inhibitor; therefore, drug–drug interactions need to be checked closely. Given its pharmacokinetic properties, therapeutic drug monitoring (TDM) is recommended as well to individualize dosage schedules, with a view to improving clinical outcomes and reducing side effects.

Posaconazole

Posaconazole is a broad-spectrum antifungal widely used for prophylaxis. It is FDA-approved for prophylaxis in invasive *Aspergillus* and *Candida* infections in patients who are at a high risk of developing these infections due to being severely immunocompromised, such as hematopoietic stem cell transplant (HSCT) recipients with graft-versus-host disease (GVHD) or those with hematologic malignancies (HM) with prolonged neutropenia from chemotherapy [32]. Posaconazole has activity in mucormycosis infections, often being used off-label and as a step-down therapy to treat invasive mucormycosis [33].

Posaconazole’s bioavailability is significantly improved when taken with food compared to fasting, up to 290% when taken with food that is high in fat for thethe oral suspension formulation. The posaconazole level was increased by only 1.5-fold when the tablet was administered with a high-fat meal than when administered in the fasted state [34]. Moreover, 20 to 30% of oral posaconazole is glucuronidated via phase 2 metabolism using UGT1A4 in the liver [33,35]. Genetic polymorphisms of *UGT1A4* alter the metabolism of posaconazole. For example, the UGT1A4*3 polymorphism is associated with the poor absorption of posaconazole oral suspensions (Table 2) [28]. Unlike other azoles, posaconazole is barely metabolized by cytochrome P450 but is a potent inhibitor of CYP3A4 [35]. Clinically relevant drug–drug interactions have been identified among drugs that are often co-administered, especially in patients with hematologic malignancies or undergoing an HSCT. For example, tacrolimus can be used for GVHD prophylaxis while the patient is on posaconazole for antifungal prophylaxis. It is recommended to reduce the dose of tacrolimus within the range of 30–50%. Concomitant dosing with H2 blockers such as cimetidine or proton pump inhibitors such as omeprazole decreases posaconazole’s serum trough levels due to reduced gastric acidity.

The multidrug resistance 1 (*MDR1*) gene encodes for P glycoprotein (P-gp), which is responsible for the bioavailability and cell toxicity limitations of a wide range of drugs. Three single-nucleotide polymorphisms (SNPs) in the coding region (C3435T, C1236T, and G2677T/A) have been related to substrate- and inhibitor-dependent functional modifications in in vitro studies and reduced expression in tissues. P-gp is a transmembrane active efflux pump for a variety of drugs, including posaconazole. P-gp expression and genotypes in regard to posaconazole pharmacokinetics were explored in 28 healthy Black and Caucasian volunteers [36]. No association was observed between any *MDR1* SNPs, including C3435T and posaconazole, in the area under the curve (AUC), a metric for total drug exposure over time [37]. Furthermore, no correlation was noted between *MDR1* mRNA levels and exposure to posaconazole. Therefore, age, gender, and race/ethnicity had no clinically relevant effects on posaconazole pharmacokinetics based on any *MDR1* SNP. In a 2006 study by Sansone-Parsons et al., no association was observed between age, gender, and race/ethnicity, which had no clinically relevant effects on posaconazole pharmacokinetics based on any *MDR1* single-nucleotide polymorphism [37]. Given these findings, there should be no difference in clinical outcomes.

However, TDM is still recommended for posaconazole. Some patients on posaconazole oral suspensions have shown low plasma concentrations; thus, TDM is needed to ensure adequate exposure, given the large variability in drug concentrations, based on high fat intake and drug–drug interactions [34].

Isavuconazole

Isavuconazole is a relatively new triazole (FDA-approved in 2015) that has broad-spectrum activity against *Candida*, *Aspergillus*, and *Cryptococcus* and mucormycosis. It is FDA-approved for invasive mucor and aspergillus infections. In addition, in real-world treatment experience with invasive fungal infections, isavuconazole was associated with a better safety profile and comparable outcomes regarding voriconazole- or amphotericin B-based regimens [38].

Isavuconazole has excellent oral bioavailability at approximately 98% [39]. As isavuconazole exhibits mild inhibition of CYP3A4, TDM is not recommended [40]. The SECURE trial was a phase 3 clinical trial that demonstrated that isavuconazole is non-inferior to voriconazole for the primary treatment of suspected invasive mold disease. The SECURE trial showed intrasubject variability in less than 30% patients [41]. This indicates that isavuconazole has predictable pharmacokinetics and therefore does not need TDM. In addition, drug–drug interactions with isavuconazole are similar to those in other azole antifungals. Given these findings, it is unlikely that there will be any consequences of race/ethnicity on isavuconazole pharmacokinetics.

Isavuconazole activity is not affected by mutations in the *MDR1* gene, which is the primary mechanism for azole resistance in *Candida* spp., leading to the overexpression of efflux pumps [42]. Since the efflux pumps encoded by *MDR1* are selective for fluconazole, isavuconazole could potentially be a useful agent for fluconazole-resistant *Candida* isolates via this mechanism [39]. 

Oteseconazole 

Oteseconazole is the latest azole to be approved in 2022, for recurrent vulvovaginal candidiasis by the FDA. Its mechanism of action is similar to those of other azoles, as oteseconazole inhibits fungal CYP51. This affects the formation and integrity of the fungal cell membrane [43]. It is important to note that this azole has a low affinity for human CYP enzymes due to its tetrazole metal-binding group. As such, this antifungal is associated with reduced drug–drug interactions and adverse events compared to other azoles [44]. However, currently, it is only approved for recurrent vulvovaginal candidiasis. It is under clinical development for the treatment of invasive and opportunistic infections. 

## 5. Clinical Outcomes and Implications

Azoles are both substrates and inhibitors for various enzymes and transporter proteins. Many drugs—most notably, proton pump inhibitors and rifamycins—can lead to significant drug–drug interactions. For example, rifamycins cause significant reductions in the serum concentrations of azoles. Pharmacokinetic studies confirm the increased maximum concentrations (Cmax) and AUCs of azoles in individuals with poor metabolism when combined with interacting drugs. Given their potential clinical impacts, future studies should explore the outcomes of combined drug–drug interactions and drug–gene interactions to enhance the safety, effectiveness, and personalization of azole therapy.

Some institutions conduct genetic testing prior to the administration of voriconazole [45,46]. By performing *CYP2C19* testing prior to the administration of voriconazole, the dosage of voriconazole can be adjusted based on the CYP2C19 phenotype, as per the CPIC guidelines. In one study by Hicks et al., utilizing this strategy resulted in higher plasma trough concentrations, and reductions in subtherapeutic concentrations were avoided in 83.8% of *CYP2C19* rapid metabolizers. This was compared to 46.2% receiving a standard dosage (*p* = 0.02) regardless of the *CYP2C19* genotype. The same study stated that *CYP2C19*-guided voriconazole dosing could prevent approximately two breakthrough fungal infections per 100 neutropenic patients with acute myeloid leukemia, resulting in modest cost savings of USD 415 per patient.

However, there are several obstacles to using *CYP2C19*-guided voriconazole dosing. These include educating providers regarding pharmacogenomics, the cost of testing and whether it is covered by insurance, the turnaround time of results, and integrating the results with the prescription of voriconazole.

## 6. Conclusions

Azole antifungals remain as the foundation for antifungal therapy due to their broad-spectrum activity and relatively favorable safety profiles. This is especially true when compared to older antifungals such as amphotericin B. Triazoles like fluconazole, voriconazole, posaconazole, itraconazole, and isavuconazole are used systemically, with progressive improvements to address resistance and broaden antifungal spectra. The pharmacokinetics of azoles are complex and influenced by factors such as the gastric pH, serum protein binding, and metabolism through cytochrome P450 enzymes in the liver. Genetic polymorphisms involving *CYP2C19* can significantly affect drug metabolism. This is significant for voriconazole, impacting both efficacy and toxicity. Consequently, therapeutic drug monitoring and genotype-guided dosing are being utilized to optimize treatment outcomes for certain azoles.

Fluconazole, by contrast, shows less variability due to genetic factors and is more influenced by renal function. Itraconazole and posaconazole, while effective, are subject to issues with absorption and bioavailability, emphasizing the role of the formulation, food intake, and pH in their therapeutic efficacy. Isavuconazole stands out for its predictable pharmacokinetics and low variability, making it less dependent on genetic or metabolic profiling. Therefore, TDM is not necessary for isavuconazole. New azoles such as oteseconazole, which promise fewer drug interactions and an improved safety profile, are now under investigation for systemic fungal infections. Directly inhaled azole therapies are also being developed for invasive fungal infections to limit systemic toxicities. Collectively, the clinical utility of azoles is increasingly being guided by pharmacogenomics, ensuring safe and effective antifungal therapy.

## Figures and Tables

**Table 1:** CPIC (2016) dosing recommendations for voriconazole treatment based on *CYP2C19* phenotype for adults [18].

CYP2C19 Phenotype	Probability of Attainment of Therapeutic Concentration with Standard Dosing	Therapeutic Recommendations	Classification of Recommendations ^a^
CYP2C19 ultrarapid metabolizer (*17/*17)	Low probability	Choose an alternative agent that is not dependent on CYP2C19 metabolism as a primary therapy in lieu of voriconazole. Such agents include isavuconazole, liposomal amphotericin B, and posaconazole. ^b^	Moderate ^c^
CYP2C19 rapid metabolizer (*1/*17)	Modest probability	Choose an alternative agent that is not dependent on CYP2C19 metabolism as a primary therapy in lieu of voriconazole. Such agents include isavuconazole, liposomal amphotericin B, and posaconazole. ^b^	Moderate
CYP2C19 normal metabolizer	High probability	Initiate therapy with recommended standard-of-care dosing. ^b^	Strong
CYP2C19 intermediate metabolizer	Modest probability	Initiate therapy with recommended standard-of-care dosing. ^b^ Higher dose-adjusted trough concentrations compared with normal metabolizers may increase the probability of adverse events.	Moderate
CYP2C19 poor metabolizer	Low probability	Choose an alternative agent that is not dependent on CYP2C19 metabolism as a primary therapy in lieu of voriconazole. Such agents include isavuconazole, liposomal amphotericin B, and posaconazole. ^b^ In the event that voriconazole is considered to be the most appropriate agent, based on all clinical implications, voriconazole should be administered preferably at a lower-than-standard dosage with careful TDM.	Moderate

^a^ Rating scheme is described in CPIC (2016) dosing recommendations for voriconazole [18]. ^b^ Further dose adjustments or selection of alternative therapy may be necessary due to other clinical factors, such as drug interactions, hepatic function, renal function, fungal species, site of infection, therapeutic drug monitoring, and comorbidities. ^c^ Recommendations based upon data extrapolated from individuals with CYP2C19*1/*17 genotype. Please see Therapeutic Recommendations based on Genotype for more information from the Clinical Pharmacogenetics Implementation Consortium (CPIC).

**Table 2:** Summary of azole metabolic pathways and genetic polymorphisms.

	Metabolic Pathways	Genetic Polymorphisms	TDM Monitoring Recommended
Voriconazole	Approximately 75% of metabolism is mediated through the CYP enzymes in the liver, while 25% occurs through flavin-containing monooxygenase [26].	*CYP2C19*, *CYP3A4*; see Table 1 for details	Yes
Fluconazole	Approximately 80% of the administered dose appears in the urine as unchanged drug. About 11% of the dose is excreted in the urine as metabolites (a glucuronide conjugate of unchanged fluconazole and a fluconazole N-oxide) [27].	*UGT2B7*, *CYP2C9*, *2C19*, and, to a lesser extent, *C3A4*	No
Posaconazole	Limited metabolism by phase 2 biotransformations via UGT enzyme pathways.	UGT1A4*3 allele polymorphisms [28]	Yes
Isavuconazole	Cytochrome P450 enzymes CYP3A4 and CYP3A5. Subsequent minor metabolism by UGT.	*CYP3A5* allele polymorphisms	No
Itraconazole	Cytochrome P450 enzyme CYP3A4. Metabolites are excreted in both urine (approximately 40%) and bile (approximately 55%).	*CYP3A4*, *CYP2C9*, or *CYP2C19* [29]	Yes

TDM: therapeutic drug monitoring, UGT: uridine 5′-diphospho-glucuronosyltransferase, CYP: cytochrome P450.
